# Robot-assisted laparoscopic radical prostatectomy for leiomyosarcoma of the prostate: A case report

**DOI:** 10.1016/j.ijscr.2025.111824

**Published:** 2025-08-20

**Authors:** Hayato Nishida, Hidenori Kanno, Hiroki Fukuhara, Sei Naito, Takanobu Kabasawa, Norihiko Tsuchiya

**Affiliations:** a Department of Urology, Yamagata University Faculty of Medicine, 2-2-2 Iida-nishi Yamagata-shi, Yamagata, Japan; b Department of Pathological Diagnostics, Yamagata University Faculty of Medicine, 2-2-2 Iida-nishi, Yamagata-shi, Yamagata 990-9585, Japan

**Keywords:** Leiomyosarcoma, Prostatectomy, Robot-assisted surgery

## Abstract

**Introduction:**

Prostate leiomyosarcomas are rare malignancies. We report the case of a 53-year-old man with locally confined prostate leiomyosarcoma treated with robot-assisted laparoscopic radical prostatectomy (RARP).

**Presentation of case:**

The patient reported frequent urination, and imaging demonstrated a well-circumscribed tumor compressing the bladder wall. Histopathological examination confirmed the diagnosis of leiomyosarcoma, with immunohistochemical positivity for α-smooth muscle actin, desmin, and h-caldesmon. Preoperative staging revealed no distant metastases, and RARP was subsequently performed. A vertical incision was made in the anterior bladder wall, facilitating the identification of the tumor arising from the ventral aspect of the prostate. Anterior bladder neck transection was achieved through intravesical resection, maintaining a safety margin of the bladder wall from the tumor. Postoperative recovery was uneventful, and the patient remained disease free and fully continent 12 months after surgery.

**Clinical discussion:**

No gold standard treatment has been established for leiomyosarcoma of the prostate; however, complete surgical resection with clear margins remains the mainstay of treatment. In cases with well-defined tumor margins, RARP with an adequate safety margin may be a viable option while preserving the postoperative quality of life and avoiding the need for a stoma.

**Conclusion:**

This case report suggests that RARP can be safely and effectively applied in the management of noninvasive prostate leiomyosarcoma.

## Highlights

• RARP with intravesical bladder neck transection safely managed noninvasive prostate leiomyosarcoma in a 53-year-old man.

## 1 Introduction

Leiomyosarcoma of the prostate is a rare tumor, accounting for less than 0.1 % of all malignant prostatic tumors (1). Unlike adenocarcinoma, which is commonly seen in prostate cancer, leiomyosarcoma of the prostate does not produce PSA and therefore often remains undetected until lower urinary tract symptoms appear. Due to the aggressive nature of the neoplasm, the rate of survival varies between 0 % and 60 %, with survival periods ranging from months to years, making the prognosis significantly poorer than that of adenocarcinoma (1). No clear guidelines have been established regarding the optimal treatment approach for locally limited leiomyosarcoma of the prostate; however, complete surgical resection is generally regarded as the treatment of choice. Robot-assisted laparoscopic radical prostatectomy (RARP) has been established as the standard surgical procedure for typical prostate cancer; however, only a few case reports have documented its use in prostate sarcoma (2). We present the case of a patient with locally limited leiomyosarcoma of the prostate treated with RARP.

This case report has been reported in line with the SCARE checklist (17).

## 2 Case presentation

**Fig. 1:**
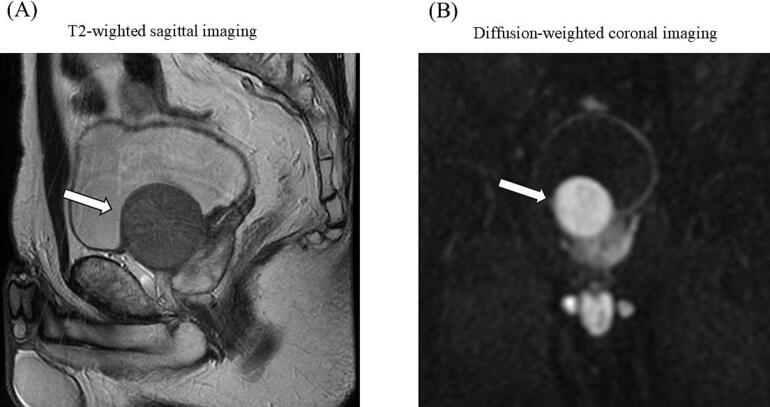
Pelvic MRI showing a well-circumscribed, homogenous tumor (arrows) with low-intensity on the T2-weighted image (A) and high-intensity on the diffusion-weighted image (B) arising from the anterior border of the prostate and compresses the bladder wall. MRI, magnetic resonance imaging.

**Fig. 2:**
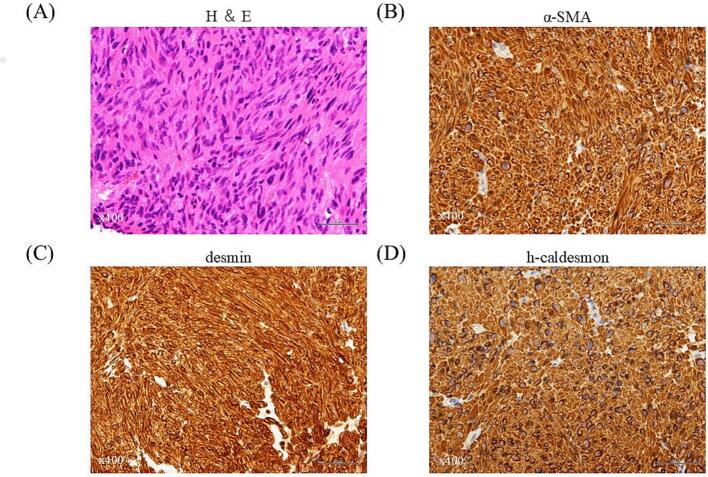
Tumor biopsy findings. (A) Hematoxylin and eosin staining shows varicella-like cells with mild nuclear atypia. Immunohistochemical analysis shows staining for (B) α-smooth muscle actin, (C) desmin, and (D) h-caldesmon in tumor cells.

A 53-year-old man presented to the urology clinic with a 4-month history of urinary frequency, occurring more than 10 times during the daytime and accompanied by a sensation of incomplete emptying and urgency. Ultrasonography revealed a large nodular mass in the prostate, and the patient was referred to our department for further evaluation and treatment. The prostate-specific antigen (PSA) level was 0.4 ng/ml. Magnetic resonance imaging (MRI) showed a well-circumscribed, homogenous tumor (4.6 × 5.0 cm) arising from the left midline of the anterior border of the prostate and compressing the bladder wall. The lesion appeared hypotense on T2-weighted images and hypertense on diffusion-weighted images (Fig. 1). A transrectal ultrasound-guided prostate biopsy was performed. Histopathological examination revealed a tumor comprising varicella-like cells with mild nuclear atypia. Immunohistochemical staining demonstrated positivity for α-smooth muscle actin (α-SMA), desmin, h-caldesmon, and Ki-67 (10 % expression), whereas AE1/AE3, CAM5.2, epithelial membrane antigen, c-kit, CD34, and S—100P were negative (Fig. 2). Based on these findings, a diagnosis of leiomyosarcoma was established. Radical surgical resection by robot-assisted laparoscopic radical cystectomy or prostatectomy without neoadjuvant chemotherapy was considered as computed tomography (CT) showed no evidence of metastasis to lymph nodes or visceral organs, and no standardized neoadjuvant chemotherapy has been established for leiomyosarcoma. Following shared decision-making among the medical staff, the patient, and his family, RARP was selected as the treatment approach.

**Fig. 3:**
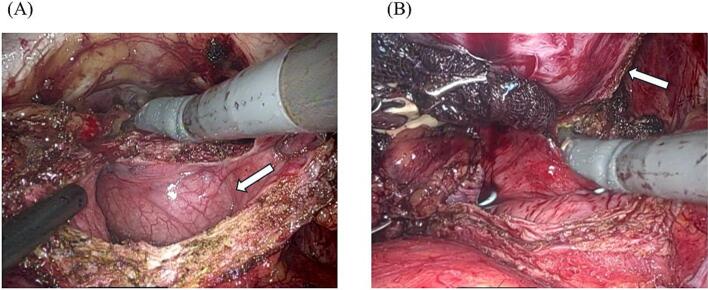
Intraoperative findings during RARP. (A) Wide anterior bladder wall incision exposing the tumor (white arrow). (B) Intravesical dissection with tumor resection and 5 mm margin from the bladder wall. RARP, robot-assisted laparoscopic radical prostatectomy.

Surgery was performed in the head-down position using the da Vinci Xi Surgical System (Intuitive Surgical, Sunnyvale, CA, USA). Following standardized pelvic lymph node dissection (PLND) and exposure of the preventral space to determine the anterior bladder neck, a vertical incision was made in the anterior bladder wall to identify the tumor arising from the ventral aspect of the prostate (Fig. 3A). Anterior bladder neck transection from the prostate was achieved through intravesical resection, maintaining a 5 mm safety margin of the bladder wall from the tumor (Fig. 3B). To transect the anterior bladder neck, it was necessary to elevate the intravesically protruding tumor to identify its boundaries. During field exposure using the third arm, in order to avoid crushing the tumor, neither the prostate nor the tumor was grasped directly. Instead, the tumor was gently elevated using gauze to secure the operative view. As the tumor was confined to the anterior lobe of the prostate, posterior bladder neck transection was performed using a standardized technique. The prostate and tumor were resected en bloc, and vesicourethral anastomosis was complete with closure of the enlarged bladder neck incision. We used 3–0 V-Loc™ barbed sutures for all the anastomoses. The final pathological diagnosis of the resected specimen was leiomyosarcoma, consistent with the biopsy findings. The surgical margin was negative. The postoperative course was uneventful except for minor leakage at the vesicourethral anastomosis. The patient was discharged 12 days after the surgery, and the transurethral catheter was removed 18 days postoperatively. As complete resection was achieved, neither postoperative radiation therapy nor adjuvant chemotherapy was administered. CT scans were performed every three months postoperatively, and the patient remained disease free and fully continent at 12 months follow-up.

## 3 Discussion

Leiomyosarcoma of the prostate, a smooth muscle sarcoma, is exceedingly rare, with only <200 cases reported worldwide. However, it is the most common primary sarcoma of the prostate in adults (1,3). The long-term prognosis remains poor due to the aggressive nature of the neoplasm and its high propensity for local recurrence and distant metastasis to the liver and lungs (4,5). Patients with leiomyosarcoma of the prostate typically present with lower urinary tract symptoms, including urinary frequency, poor stream, hesitancy, urinary urgency, voiding difficulty, painful ejaculation, and hematuria (1). As the tumor progresses, perineal pain and weight loss may occur; however, due to the nonspecific clinical features, the tumor is often overlooked or misdiagnosed as benign prostatic hyperplasia (3,6,7). Diagnosis is typically established through transrectal ultrasound-guided needle biopsy or transurethral resection (8). Pathological diagnosis is based on the identification of cellular proliferation of spindled neoplastic cells resembling smooth muscle cells, exhibiting nuclear atypia and increased mitotic activity, with occasional epithelioid cells (4,7). Most cases of leiomyosarcoma of the prostate tend to have high-grade features on microscopic examination associated with areas of viable tumor which comprise hypercellular, intersecting bundles of eosinophilic, spindle-shaped cells that have variable degrees of nuclear mitotic activity as well as nuclear atypia (1). Neoplastic cells typically express vimentin, α-SMA, and desmin, whereas S-100, cluster of differentiation 34 (CD34), and PSA are negative (4). The local extent and distant spread of the disease can be assessed by CT or MRI, which provide clear delineation between neoplastic and nonneoplastic tissues (4).

No gold standard treatment has been established for leiomyosarcoma of the prostate; however, complete surgical resection with clear margins remains the mainstay of treatment (3). Multimodal treatment approaches, including preoperative or postoperative radiation therapy and neoadjuvant or adjuvant chemotherapy with anthracycline-based regimens combined with alkylating agents, have been reported (1,9). In our patient, preoperative chemotherapy and radiation therapy were not administered, but immediate surgery was performed. A distinct boundary between the tumor and normal tissue was identified, with no evidence of infiltration, allowing complete resection without the need for preoperative therapy. Moreover, no established regimen for preoperative radiation or neoadjuvant chemotherapy reliably achieves tumor shrinkage. This poses a risk of missing the opportunity for complete surgical resection given the tumor's rapid progression.

RARP has become the standard surgical procedure for primary localized prostate cancer and is now also performed in patients with high-risk prostate cancer owing to its optimal outcomes (2). However, RARP for prostate sarcoma has been rarely reported. To our knowledge, this is the first reported case of RARP performed for leiomyosarcoma of the prostate. In total, only eight patients who underwent RARP for prostate sarcoma, including stromal sarcoma, stromal tumor of uncertain malignancy, and undifferentiated pleomorphic sarcoma, have been documented since Choi et al. first reported RARP for prostatic stromal sarcoma ([10], [11], [12], [13], [14], [15], [16]) (Table 1). No standardized treatment strategy has been established for prostate sarcoma, including leiomyosarcoma. Complete excision with negative surgical margins is preferred for surgical resection. Radical prostatectomy has been previously reported as a treatment for localized leiomyosarcoma of the prostate without obvious invasion into surrounding tissues including the bladder (3,6). In the present case, the tumor had well-defined margins, and we determined that complete resection could be achieved by resection up to the bladder neck, in accordance with the principles of radical prostatectomy. There were no significant intra-abdominal adhesions or comorbidities that would contraindicate RARP, such as those interfering with the Trendelenburg position. Therefore, we selected RARP to perform the surgery in a less invasive manner. Among the eight documented patients, recurrence in two: rectal invasion and extracapsular extension of the prostate (10,13) (Table 1). Radical cystectomy or total pelvic exenteration, including prostatectomy, is more feasible than prostatectomy alone for tumors invading the surrounding tissue. However, in cases with well-defined tumor margins, such as in our patient, radical prostatectomy with an adequate safety margin may be a viable option while preserving the postoperative quality of life and avoiding the need for a stoma.

**Table 1:** Case reports of RARP for sarcoma of prostate.

Author	Year	Age (years)	Pathology	Lymph node dissection	Vertical incision for anterior wall of the bladder	Follow-up	Outcome
Choi et al.	2014	29	Stromal sarcoma	Performed	Not performed	45 days	Recurrence
Mao et al.	2016	32	Stromal sarcoma	Not performed	Performed	6 months	No recurrence
Agarwal et al.	2018	7	Embryonal Rhabdomyosarcoma	Performed	Not performed	3 months	No recurrence
Iwashita et al.	2020	27	Undifferentiated pleomorphic sarcoma	Performed	Not performed	18 months	Recurrence and death
Suzuki et al.	2020	60	STUMP	Not performed	Not performed	6 months	No recurrence
Dokubo et al.	2023	60	STUMP	Not mentioned	Not mentioned	1.6 years	No recurrence
Dokubo et al.	2023	65	STUMP	Not mentioned	Not mentioned	4.8 years	No recurrence
Zhu et al.	2024	Early 40s	STUMP	Not mentioned	Not mentioned	Not mentioned	Not mentioned
This case	2025	53	Leiomyosarcoma	Performed	Performed	1 year	No recurrence

RARP, robot-assisted laparoscopic radical prostatectomy; STUMP, stromal tumor of uncertain malignant potential.

In the present case, PLND was performed; however, its effectiveness in prostatic sarcomas remains unclear. Prostatic sarcomas, including leiomyosarcoma, predominantly metastasize via the hematogenous route, and lymph node involvement is considered rare. Nonetheless, a few cases of prostatic leiomyosarcoma with lymph node metastasis have been reported (1). Similar to adenocarcinoma, PLND may have prophylactic or diagnostic value in selected cases of sarcoma, and lymph node dissection has been performed with some frequency in previously reported RARP cases for prostatic sarcomas (10,12,13). As the standard technique for PLND in RARP has been well established and can be safely performed, its selective application is considered clinically reasonable.

## 4 Conclusion

We encountered a case of locally limited leiomyosarcoma of the prostate treated with RARP. Under precise imaging, RARP proved to be a feasible and effective surgical option for prostatic leiomyosarcoma without periprostatic invasion. Opening the bladder lumen and performing bladder neck resection while visually identifying the tumor margin were effective approaches in cases where the tumor protruded into the bladder lumen.

## Author contribution

H.N., H.K., and H.F. performed the experiments. H.N., H.K., H.F., S.N., T.K., and N.T. made substantial contributions to the conception and design of this case report. H.N. drafted the manuscript, and H.K., H.F., S.N., T.K., and N.T. critically revised it. N.T., as the chairperson of our department, provided supervision and guidance throughout the process. H.N., H.K., H.F., S.N., T.K., and N.T. have read and agreed to the final version of the manuscript.

## Consent

Written informed consent was obtained from the patient for publication of this case report and accompanying images. A copy of the written consent is available for review by the Editor-in-Chief of this journal on request.

## Ethics approval

This study was conducted in accordance with the principles of the Declaration of Helsinki. According to the Japanese ethical guidelines, case reports do not require review by the ethics board of our institution.

## Guarantor

Hayato Nishida

## Research registration number

Not applicable.

## Declaration of Generative AI and AI-assisted technologies in the writing process

During the preparation of this work the author used ChatGPT in order to English translation only in Introduction and Discussion sections. After using this tool/service, the corresponding author reviewed and edited the content as needed and takes full responsibility for the content of the publication.

## Funding

The authors did not receive financial support for this study.

## Declaration of competing interest

Authors declare no Conflict of Interests for this article.
